# Assessing the regional climate impact on terrestrial ecosystem over East Asia using coupled models with land use and land cover forcing during 1980–2010

**DOI:** 10.1038/s41598-020-59503-4

**Published:** 2020-02-13

**Authors:** Fuqiang Cao, Li Dan, Zhuguo Ma, Tao Gao

**Affiliations:** 1 School of Geoscience, Shanxi Normal University, Linfen, Shanxi 041004 China; 2 Key Laboratory of Regional Climate-Environment for Temperate East Asia, Institute of Atmospheric Physics, Chinese Academy of Sciences, Beijing, 100029 China; 3 College of Urban Construction, Heze University, Heze, 274000 China; 4 State Key Laboratory of Numerical Modeling for Atmospheric Sciences and Geophysical Fluid Dynamics, Institute of Atmospheric Physics, Chinese Academy of Sciences, Beijing, 100029 China

**Keywords:** Climate change, Environmental sciences, Environmental impact

## Abstract

The coupled model AVIM-RIEMS2.0 is employed to examine the effects of climate change on the terrestrial ecosystem over East Asia during three decades since the 1980s. The vegetation parameters present significantly different responses to climate change in subregions, since the effects of climate change trigger seasonal signals on land surface processes at the regional scale. In the 1980s, the increasing temperature and rainfall lead to a decrease in biomass and leaf area index (LAI) in winter, but a slight increase in net primary productivity (NPP) over China. However, summertime precipitation shows interval changes of cyclic increase-decrease pattern over eastern China, and the similar pattern also occurs for the variations in biomass and LAI. In the 1990s, the temperature and precipitation over the most regions in East Asia demonstrate the opposite changes compared to the 1980s, which results in converse variations in LAI and vegetation carbon flux. In the 2000s, biomass and LAI in the mid-lower reaches of Yangtze River basin and southeast coastal regions exhibit the same changes as precipitation in winter, and NPP shows a similar response to temperature. The biomass and LAI show consistent responses to regional climate change in summer, while different responses are seen for NPP. In general, climate change had a great impact on the vegetation in the 1990s, which produced the remarkable influences on LAI and biomass in winter and the significant impacts on NPP in summer. Over the regions affected significantly by East Asian monsoon, e.g. South China, the terrestrial ecosystem displays a roughly consistent response to regional climate change.

## Introduction

During the past century, global climate change, particularly the temperature increase has greatly impacted the hydrological processes and water circulation^1–4^. These variations not only result in disastrous consequences, but also have close linkages with variability of vegetation parameters^5,6^. Vegetation changes are usually represented by land use and land cover change (LULCC)^5^. And LULCC often illustrates the maps of land surface process that are associated with climate change and human activities. In the context of climate change, the spatial distribution of LULCC is one of essential prerequisites for developing and planning the management of natural resources.

Investigating LULCC and climate change are two important global development projects of the Intergovernmental Panel on Climate Change Fifth Assessment Report (IPCC AR5), which have attracted increasing concern during recent decades. The effects of LULCC on regional climate changes (e.g., temperature and precipitation) can be examined in terms of different biogeophysical and biogeochemical processes. Biogeophysical mechanisms can affect the water cycles and surface energy by modifying the physical processes at land surface^7,8^. Biogeochemical impacts alter the carbon cycles through the absorptions or emissions of atmospheric greenhouse gasses, and thus influence the regional climate system^9,10^. The interaction between regional climate change and LULCC is sophisticated process, which reflects the interactions between hydrological, geological, human activities and climate change^11–13^. However, existing studies focusing on the interaction between climate change and LULCC are mainly concentrated on the effects of the LULCC on regional climate change^5,14–19^, very few researches have been carried out on the issues about how the regional climate change influences LULCC.

LULCC is a forcing impact on the surface heat fluxes, which leads to the climate changes at different spatial and temporal scales, and vice versa^20^. Sivakumar^21^ analyzed the two-way interactions between climate and desertification, and concluded that the variations in land degradation over the drylands mainly result from the climatic changes and human activities. During 1986–2003 in India, the enhanced increasing trend in meteorological conditions, especially temperature and precipitation, results in the increasing wasteland, uncultivated land and water body, but the decreases occur in the cultivated land. In addition, the decrease in forest may be attributed to the increase in built-up land and population^22^. In sub-humid, semi-arid and arid regions, the land degradation resulted from the loss of vegetation cover is largely caused by climate change, which can impact species succession, hydrologic cycles and soil quality, and therefore expands the dryland areas^23–26^. Alves *et al*.^26^ found that climate change plays an important role on the land degradation (desertification) in Brazil from 1950 to 2013. Rahman^23^ reported that total rainfall has significant effects on the increases in agricultural land use diversity over Bangladesh during 1948–2008.

In China, Feng *et al*.^24^ conducted the quantitative assessments of interactions between climate change and desertification through long-term monitoring from 1983 to 2012, and suggested that climate change accounts for roughly 46.6% of the effects on normalized difference vegetation index (NDVI) and desertification. Shi and Chen^27^ pointed out that temperature exhibits a stronger correlation with NDVI compared to rainfall by analyzing the linkage between LULCC and climate change in Yunnan Province. The influences of climate change on land use change over Poyang Lake district are discernible during 1985–2035, and the global warming may even amplify these complex interactions. Thus, the variations in forest, grassland and cropland are more sensitive to climate change than unused land^19,28^. With ongoing climate change, desertification shows an accelerated trend under the RCP 8.5 scenario over middle and northern Middle Asia, Mongolian Plateau and northwestern China, except for Xinjiang. Moreover, the RCP 8.5 scenario triggers a more strengthened trend of desertification in comparison with the RCP 2.6 scenario^29^. Based on 30-year NDVI, Liu *et al*.^30^ confirmed the importance of temperature on the phenological processes in China, and also suggested the incorporation of precipitation and temperature into phenological models to improve their performances.

Through reviewing literature on relevant responses of ecosystems to extreme climate events, Frank *et al*.^31^ demonstrated that extreme temperature (precipitation) events have widespread influences on terrestrial carbon cycle, and further pointed out that the underlying processes of the climate change impacts on LULCC are more complex and still poorly understood. Moreover, considering these complicated processes, traditional methodologies, such as linear programming, statistical analysis and geographical information system can hardly explore the physical causes of the impacts of regional climate change^19,32^. In this study, we utilize the numerical model AVIM-RIEMS2.0 containing the dynamic vegetation process^33,34^, to investigate the interaction between regional climate change and vegetation carbon flux as well as LAI over East Asia. The rest of paper is organized as follows: The experimental design and methodology are described in section 2. Section 3 describes the simulation results. The climate-vegetation changes in typical regions over China are provided in section 4, followed by conclusions in section 5.

## Experimental Design and Methodology

### Model setup and configuration

**Figure 1:**
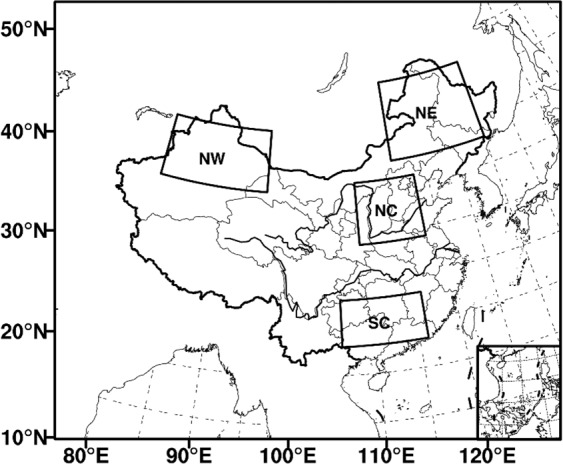
The simulation domain and typical subregions. NE, NC, SC, and NW denote the Northeast, North China, South China, and Northwest, respectively. The maps in the figure were created by C.F. using NCL 6.5 (http://www.ncl.ucar.edu/).

By introducing the LULCC datasets (described in section 2.2), the AVIM-RIEMS2.0 coupled model was driven by the National Centers for Environmental Prediction/Department of Energy (NCEP/DOE) Reanalysis 2 datasets^35^ (hereafter as NCEPII) with temporal resolution of 6 hours. We conducted four simulations from *January 1, 1979 to March 1, 1985* (*B*_0_: as a control test), *January 1, 1987 to March 1, 1993* (B_1_), *January 1, 1997 to March 1*, 2*00*3 (*B*_*2*_), and *January 1, 2007 to March 1, 2013* (*B*_*3*_). The first year of each simulation was not used in analysis due to the model spin up. The model domain had 105 (zonal) × 91 (meridional) grid points, and was centered at 37°N and 102^◦^E with a spatial resolution of 60 km, 16 atmospheric levels, 12-grid-point buffer and a 120 s time step and six-hour output. We selected East Asia, including China, as the model domain (Fig. 1), since the discernible variations occurred during last decades^36–38^.

### Methods

We obtained the vegetation types and land use datasets from 1981 and incorporated them in the AVIM-RIEMS2.0 coupled model. The data was provided by *973 project groups* (grant No. 2011CB952000). The four stages (B_0_, B_1_, B_2_, and B_3_) of NCEPII datasets were used to provide initial and boundary conditions, which were considered as the climate change in three periods (B_1_ minus B_0_ as 1980s, B_2_ minus B_1_ as 1990s, and B_3_ minus B_2_ as the 2000s). The five-year simulation results of four stages were averaged, respectively, to examine the regional climate effects on LULCC through assessing the impacts of temperature and precipitation on the changes in leaf area index (LAI), biomass and net primary production (NPP). The temperature and precipitation are the important climate components and key influencing factors of land use and land cover^9,39,40^. Therefore, the regional climate change was evaluated by the differences of temperature and precipitation among three periods. We selected winter (December, January, February, DJF) and summer (June, July, August, JJA) as the representative of typical seasons, since these two seasons had prominent changes in vegetation^6,41^. The comparative analyses of the simulations in typical subregions were provided in sections 3 and 4.

## Results

### Temperature

**Figure 2:**
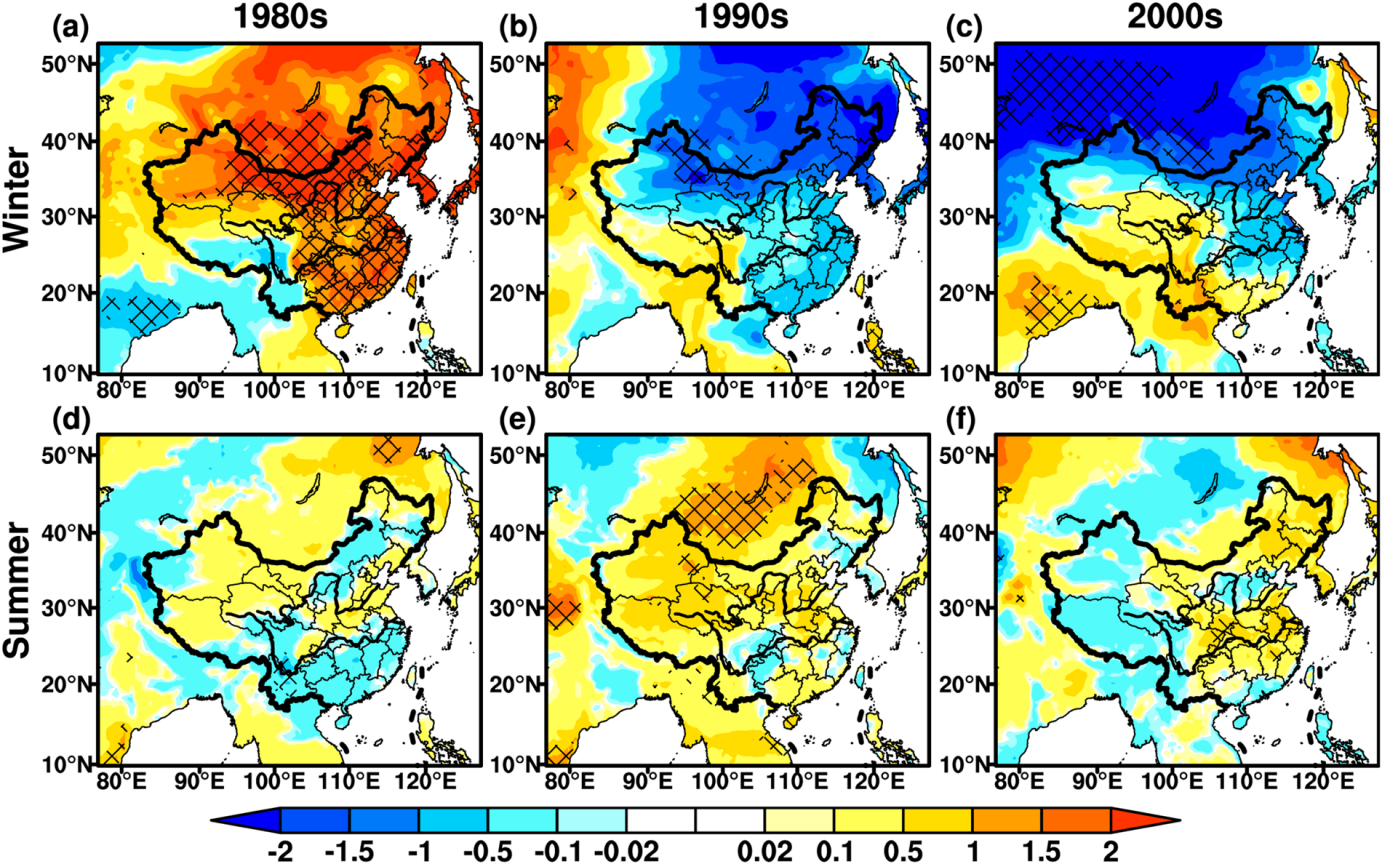
Monthly mean temperature differences (Unit: °C). (**a–c**) Represent differences in the 1980s, 1990s and 2000s; (**d–f**) are the same as (**a–c**), but for summer. Hatching denotes regions with the significance at 95% confidence level. The maps in the figure were created by C.F. using NCL 6.5 (http://www.ncl.ucar.edu/).

Variability of temperature has a crucial role on the regional climate and environment^42^. Figure 2 shows the distribution of monthly mean temperature differences. In the 1980s, the wintertime temperature exhibits an evident increase over the most parts of East Asia, with the maximum up to 2.0 °C. Especially, the temperature differences with the statistical significance exceeding 95% confidence level are found over central-east China, Inner Mongolia, Mid-southern Mongolia and Korean Peninsula (Fig. 2a). The amplitude of summertime temperature differences is weakened compared to the winter, with the range of ±0.5 °C in vast regions. The cooling regions, which mainly distribute in the south of Yangtze River basin (YRB) and agro-pastoral transition zone, increase remarkably in the 1980s (Fig. 2d). Around the 1990s, the wintertime temperature almost displays converse changes in comparison with the 1980s (Fig. 2a,b), and strong cooling regions occur in northeastern East Asia (Fig. 2b). The behaviors of summertime temperature are generally opposite to winter, and significant warming changes appear over the most parts of East Asia (Fig. 2b,e). In Mongolia, the maximum increased summertime temperature with 95% confidence level is up to 1.5 °C. The summertime temperature displays a cooling change in Sichuan-Chongqing region over southwest China (Fig. 2e). In the 2000s, the wintertime temperature exhibits the similar changes to the 1990s, with stronger cooling regions in North China and warming changes in southern East Asia. The changing range of wintertime temperature is roughly ±1.5 °C, and the maximum decrease up to 2.0 °C is seen in northeastern East Asia (Fig. 2c). However, the amplitude of summertime temperature changes weakens to ±0.5 °C, and the warming regions are mainly located over the border between Mongolia and North China, Korean Peninsula, Northeast China and mid-lower reaches of YRB (Fig. 2f).

### Precipitation

**Figure 3:**
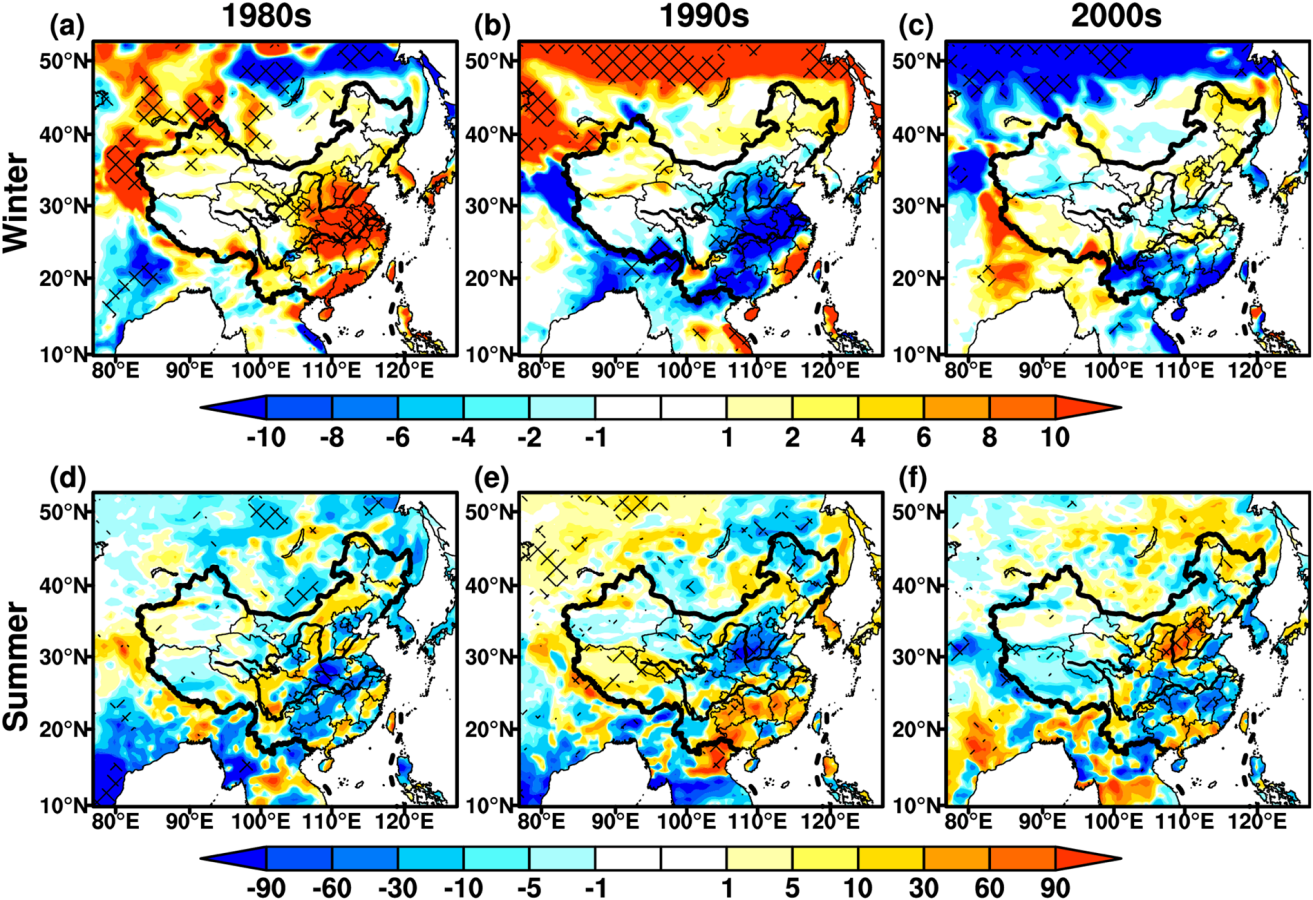
Same as Fig. 2, but for monthly precipitation (Unit: mm). The maps in the figure were created by C.F. using NCL 6.5 (http://www.ncl.ucar.edu/).

The variations in rainfall have a larger uncertainty in comparison with temperature^43,44^. Rainfall in East Asia mainly concentrates in summer owing to the effects of monsoon climate^45^, summertime precipitation has larger changes than winter (Fig. 3). In the 1980s, wintertime rainfall increases markedly in southeastern and northwestern East Asia, with maximum discrepancies up to 10 mm (Fig. 3a). Some regions present significant differences exceeding 95% confidence level, such as the mid-lower reaches of YRB and western Mongolia. The summertime precipitation decreases significantly in Henan province, with the maximum value of 90 mm exceeding 95% confidence level. Meanwhile, a distribution pattern of cyclic increase-decrease for summertime rainfall occurs from south to north over China (Fig. 3d). In the 1990s, the wintertime rainfall exhibits roughly opposite changes to the 1980s in the Yellow River basin, YRB and southwestern China, especially in the mid-lower reaches of YRB (Fig. 3b). Summertime precipitation around the 1990s also shows a generally opposite change in contrast with the 1980s over south of the mid-lower reaches of YRB, Northwest China and the most of regions in Mongolia. The summertime rainfall decreases significantly at the 95% confidence level in North China and forms a low value center at the junction of Shaanxi, Shanxi and Henan provinces, but increases in the southern portion of mid-lower reaches of YRB and Qinghai-Tibet plateau (Fig. 3e). In the 2000s, the variations in wintertime precipitation are weaker than other periods. In vast areas of East Asia, the wintertime precipitation in the 1990s has an opposite behavior to the 1980s, particularly in North China and southeast coastal regions (Fig. 3c). The summertime rainfall in the 2000s over most regions of East Asia illustrates an opposite change compared to the 1990s, with significant increases at the 95% confidence level in North China (Fig. 3f). In general, the changes in precipitation pattern are significant during three periods, while the variations in rainfall intensity are also remarkable.

### LAI

**Figure 4:**
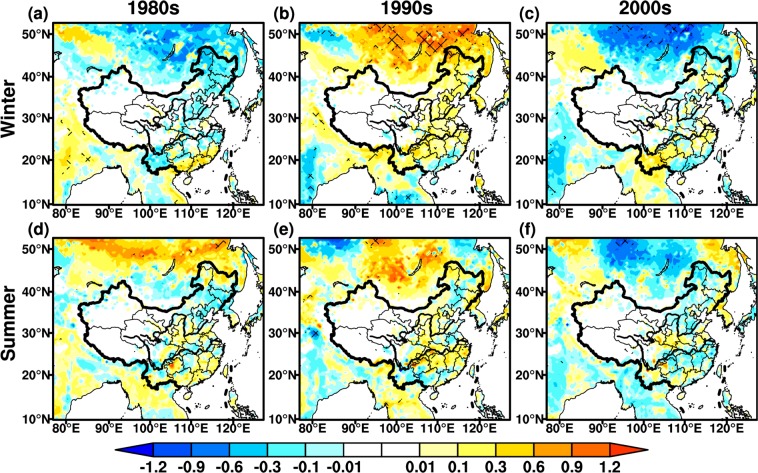
Same as Fig. 2, but for monthly mean LAI (Unit: m^2^·m^−2^). The maps in the figure were created by C.F. using NCL 6.5 (http://www.ncl.ucar.edu/).

LAI is the critical parameter for land surface and climate modeling studies^33,46^, which is a bridge between the physical and physiological processes of the AVIM-RIEMS2.0. Around the 1980s, the wintertime LAI decreases in most regions of East Asia, while an increase is found in southern coastal regions over China, significant at the 95% confidence level (Fig. 4a). The summertime LAI increases evidently over East Asia, with the maximum up to 1.2 m^2^·m^−2^. The areas with LAI differences being significant at the 95% confidence level also extend remarkably in summer (Fig. 4d). In the 1990s, the wintertime LAI differences exhibit a significant increase, especially in North China and Mongolia, with the maximum increase of 1.2 m^2^·m^−2^. Whereas the opposite changes are seen around the 1980s (Fig. 4a,b). The variations in summertime LAI are analogous to winter over East Asia, but the changing amplitude weakens slightly (Fig. 4d,e). In the 2000s, wintertime LAI shows an opposite change compared to the 1990s in North China, while the consistent variations occur in the mid-lower reaches of YRB and southern coastal regions over China. The change in wintertime LAI is relatively weaker in vast regions, with range of ±0.8 m^2^·m^−2^ (Fig. 4c). The pattern of summertime LAI differences is generally similar to winter, and the opposite changes are found in Korean Peninsula, mid-lower reaches of YRB and Guangdong province (Fig. 4f).

### Biomass

**Figure 5:**
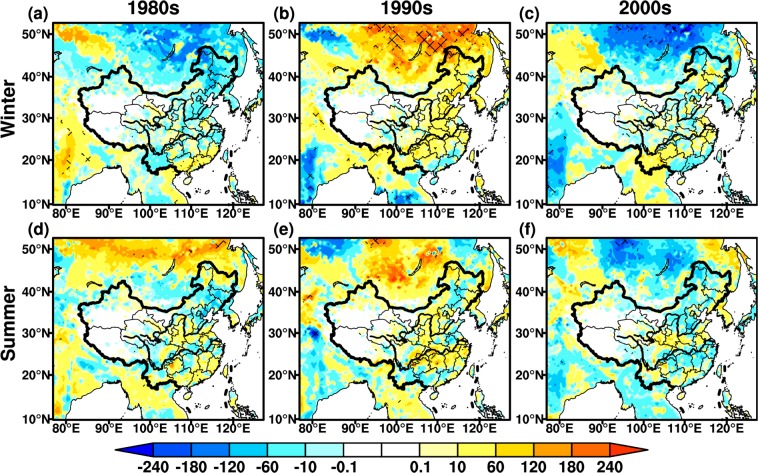
Same as Fig. 2, but for biomass (Unit: g·C·m^−2^). The maps in the figure were created by C.F. using NCL 6.5 (http://www.ncl.ucar.edu/).

Biomass is one of the basic variables for the quantitative study of the interaction between climate change and vegetation^47,48^. In general, wintertime biomass differences show more evidently seasonal features than summer (Fig. 5). In the 1980s, the biomass decreases significantly in winter, with the maximum change up to −240 g·C·m^−2^ in North China, and some areas are statistically significant at the 95% confidence level (Fig. 5a). The wintertime biomass increases remarkably over northern East Asia and southern coastal regions in China, this is similar to LAI changes (Figs. 4a and 5a). The summertime biomass increases significantly in China, but the differences weaken with range of ±100 g·C·m^−2^. The decrease in summertime biomass is principally discovered in the mid-lower reaches of YRB, which is analogous to LAI changes in corresponding periods (Figs. 4d and 5d). In the 1990s, wintertime biomass exhibits a generally opposite change to the 1980s (Fig. 5a,b). The biomass differences decrease significantly in North China and Mongolia, with the maximum values greater than 240 g·C·m^−2^, exceeding the 95% confidence level (Fig. 5a). The summertime changes in biomass are similar to winter, while the magnitude of variations in summer is relatively small, not significant at 95% confidence level. The summertime biomass declines in central portions of North and Southwest China, consistent with changes for LAI (Figs. 4e and 5e). In the 2000s, the biomass decreases in both winter and summer over vast regions, significant at the 95% confidence level (Fig. 5c,f). The wintertime biomass presents opposite changes in the mid-lower reaches of YRB, with maximum value up to 60 g·C·m^−2^, while the same pattern with opposite changes is found in summer.

### NPP

**Figure 6:**
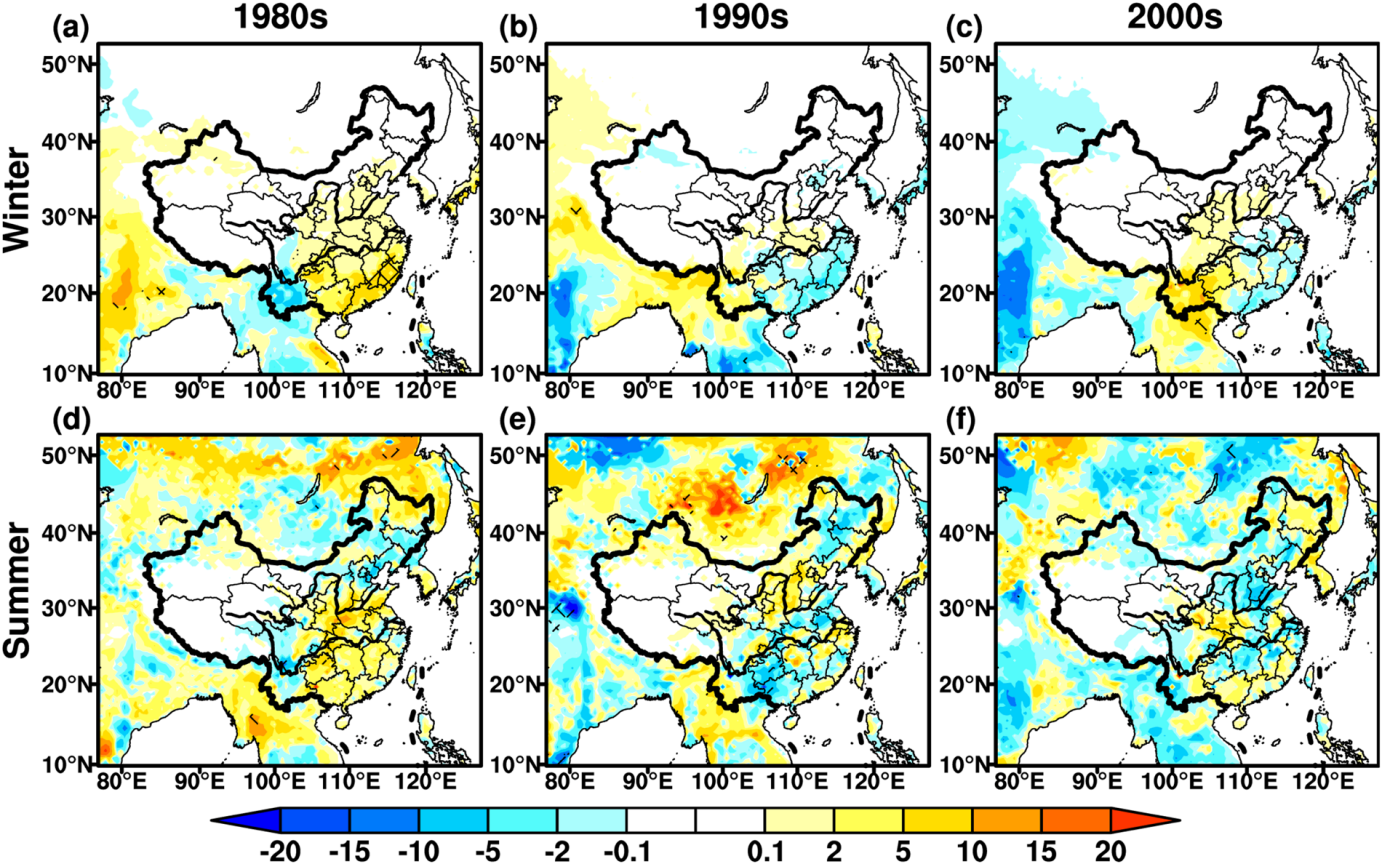
Same as Fig. 2, but for net primary productivity (Unit: g·C·m^−2^). The maps in the figure were created by C.F. using NCL 6.5 (http://www.ncl.ucar.edu/).

NPP is net absorption of carbon by vegetation, representing the energy source of human activities^33,49^. It is also an important index to evaluate plant community productivity in natural environment^50^, reflecting the responses of ecosystem to environmental and climatic conditions^51,52^. The seasonal features of NPP are more distinct in winter than summer (Fig. 6), while this phenomena are not discernible for biomass. In the 1980s, an increase in wintertime NPP is mainly distributed in central-east China, especially in South China with the maximum up to 10 g·C·m^−2^. The wintertime NPP has a pronounced decline in Southwest China (Fig. 6a). An increase in summertime NPP is mainly located in north of Northeast China, mid-lower reaches of YRB and South China, with maximum up to 15 g·C·m^−2^, they are significant at the 95% confidence level, while a slight decrease is seen in few regions (Fig. 6d). In the 1990s, the changes in wintertime NPP are relatively weaker than summer, with range between ±5 g·C·m^−2^. The opposite changes present around the 1980s, particularly in south of the mid-lower reaches of YRB (Fig. 6b). Summertime NPP changes enhance significantly, exceeding 95% confidence level in some areas of Yunnan province and Mongolia (Fig. 6e). NPP around the 1990s also show opposite variations in summer compared to 1980s over East Asia. In the 2000s, the wintertime NPP changes are analogous to the 1990s, while the increase in NPP intensities is more strengthened around the 2000s. The wintertime NPP increases remarkably in Southwest China with the maximum values up to 15 g·C·m^−2^ (Fig. 6c). In summer, the NPP displays a relatively weaker decrease with the range between ±10 g·C·m^−2^. The variations in summertime NPP are opposite to the 1990s over Mongolia, North and South China (Fig. 6e,f), this is similar to LAI and biomass in the corresponding periods.

## Discussion

### Vegetation carbon flux and vegetation morphology over East Asia

The variations in vegetation carbon flux and morphology manifest the seasonal and regional features during three selected periods. In the 1980s, same as the changes in wintertime temperature and precipitation, an increase in wintertime NPP appears in the most regions of East Asia. However, the wintertime LAI and biomass exhibit pronounced declines over East Asia, which might cause a decrease in vegetation respiration and contribute to the NPP increase. Summertime LAI and biomass display cyclic increase-decrease from south to north over East China, dominated by the influences of summertime rainfall. The analogous interval distribution is also seen for summertime NPP, even though its variability is insignificant. In the 1990s, the positive changes in wintertime LAI and biomass are mainly located in East China, except for some southeast coastal regions, where the roughly opposite changes are seen for wintertime temperature, precipitation and NPP. With the influences of temperature and precipitation, the corresponding summertime LAI, biomass and NPP show similar changes over vast regions of East Asia in the 1980s. In the 2000s, the wintertime LAI, biomass and NPP exhibit a prominent decline due to the effects of temperature and precipitation, while an increase in NPP is found over Southwest China. In summer, the vegetation parameters show an analogous behavior to winter, but the wintertime increase shifts into summertime decrease in the south of mid-lower reaches of YRB. In general, the variations in LAI and biomass display the same changing behaviors derived from the influences of climatic factors, while these are different from the changes in NPP. The impacts of climate change exert discrepancies on vegetation carbon flux and vegetation morphology. For example, an increase in precipitation causes a decrease in LAI, biomass and NPP over North China, whereas the opposite phenomena occur in the MLYRB located in South China.

### The climate-vegetation changes in China

According to the distribution of climate zones as well as dry and wet soil conditions, China can be divided into four typical subregions: Northeast China (NE), North China (NC), South China (SC), and Northwest China (NW), which are marked with rectangles in Fig. 1. These divided subregions are synthetically determined based on the National Assessment Report on Climate Change (CMA 2014). From the perspective of five-year month averages in four typical subregions, we further analyze the responses of vegetation parameters to regional climate change.

#### Northeast China

NE is located in the middle-high latitudes, and is more sensitive to climate change^53,54^. Table 1 shows that the temperature increases in the 1980s, and the precipitation decreases (increases) in summer (winter). Consequently, biomass (NPP) decreases (increases) around the 1980s and LAI increases (decreases) in summer (winter). In the 1990s, rainfall (temperature) has a large decline in summer (winter), and shows an increase in winter (summer). Both LAI and biomass increase in summer and winter, particularly for biomass exhibiting a pronouncedly strengthened increase. While NPP decreases both in summer and winter. Precipitation increases in the 2000s, and temperature decreases in winter. Correspondingly, both LAI and biomass increase in summer and winter, but NPP has an opposite behavior. In short, changes in LAI and biomass are roughly consistent with rainfall variability in the 1990s. Nevertheless, consistent variations are found between NPP and precipitation around the 2000s.

**Table 1:** Five-year regional average of climatic elements and vegetation parameters in Northeast China.

Date		Temperature (°C)	Precipitation (mm)	LAI (m^2^·m^−2^)	Biomass (g·C·m^−2^)	NPP (g·C·m^−2^)
1980s	Summer	0.02	−6.7	0.03	−3.3	2.53
	Winter	1.63	1.3	−0.22	−160.6	0.04
	Year	0.89	−35.7	−0.10	−28.1	0.18
1990s	Summer	0.24	−12.4	0.04	19.5	−2.45
	Winter	−1.85	5.4	0.23	160.5	−0.03
	Year	−0.73	2.8	0.14	30.7	0.42
2000s	Summer	0.43	2.6	−0.06	−45.0	0.63
	Winter	−1.13	5.4	−0.05	−23.1	0.01
	Year	0.04	20.2	−0.03	−3.5	0.33

The “−” sign indicates a decrease.

**Figure 7:**
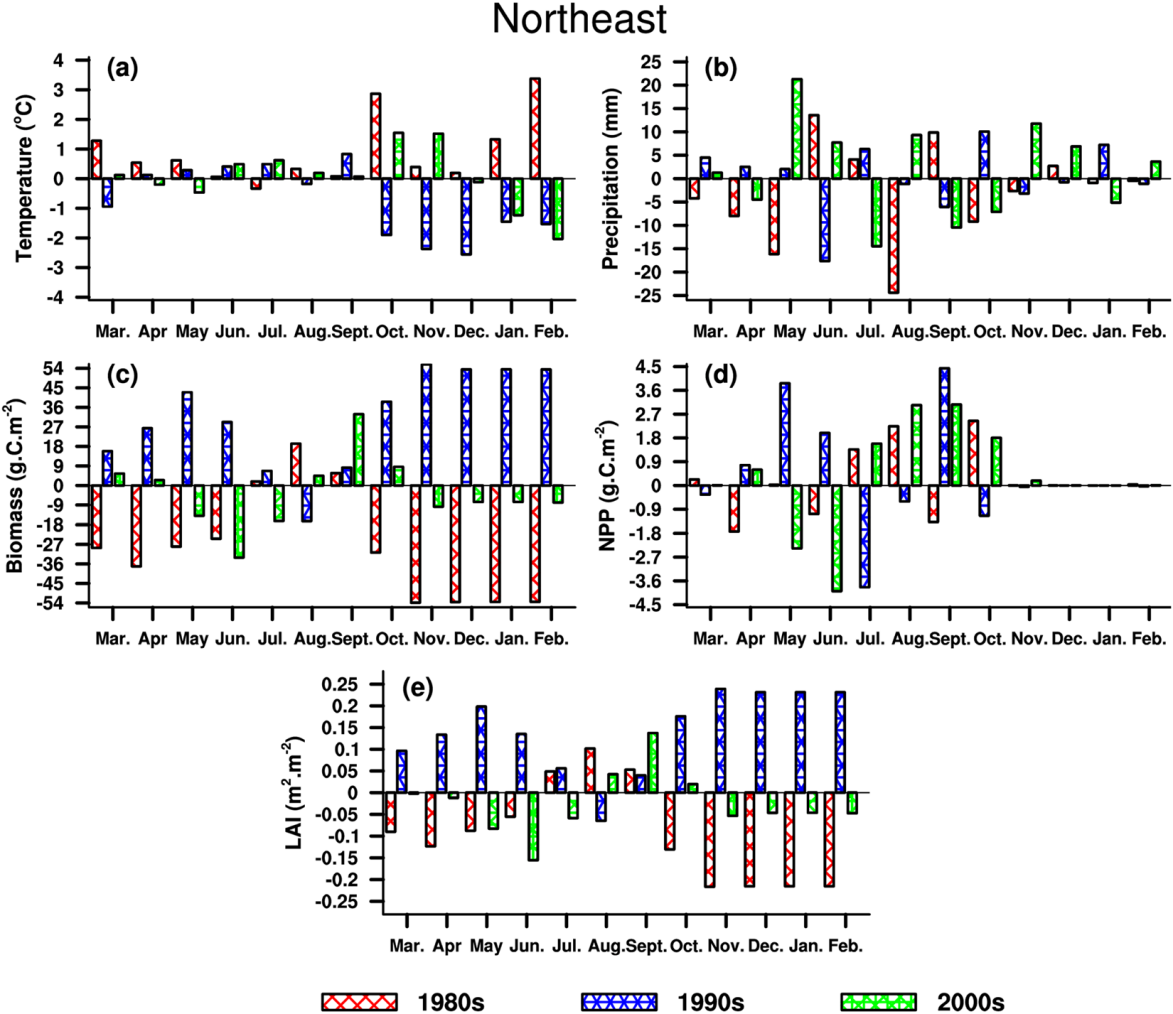
Five-year average differences of climate and vegetation in Northeast China. The maps in the figure were created by C.F. using NCL 6.5 (http://www.ncl.ucar.edu/).

Figure 7 illustrates that there are great differences of changes in vegetation parameters during three periods. In the 1980s, both biomass and LAI show a decline derived from the effects of the increasing (decreasing) temperature (precipitation), with larger intensities appearing in winter half-year. Unlike behaviors of LAI and biomass, the NPP change is stronger in summer half-year, and exhibits an analogous feature to temperature in most months, while it is contrary to precipitation. In the 1990s, the vegetation has an approximately opposite change to the 1980s, which might occur due to the opposite changes in climate during two periods. In the 2000s, the vegetation presents weaker changes, because the variations in temperature and precipitation are smaller compared to other two periods. Both LAI and biomass decline slightly, and the NPP mainly increases in summer half-year, consistent with changes in temperature. The changes in vegetation are principally impacted by temperature in NE, while the change in NPP is subjected to the variability of rainfall in growing seasons.

#### North China

NC is located in boreal temperate zone, where the vegetation exhibits seasonal variations corresponding to monsoon climate. As shown in Table 2, Temperature increases around the 1980s, and precipitation decreases (increases) in summer (winter). Both LAI and biomass decrease in summer and winter, while the opposite behaviors are found for NPP. In the 1990s, temperature decreases (increases) in winter (summer), whereas the rainfall has a large decline both in summer and winter. Rainfall increases in the 2000s, and temperature increases (decreases) in summer (winter). Consequently, three vegetation parameters (LAI, NPP and biomass) decrease (increase) in summer (winter), and the biomass has a decline in winter. Particularly, an increase in temperature coincides with a decrease in LAI and biomass around the 1980s, while a decline in rainfall is consistent with an increase in LAI and biomass around the 1990s.

**Table 2:** Same as Table 1, but for North China.

Date		Temperature (°C)	Precipitation (mm)	LAI (m^2^·m^−2^)	Biomass (g·C·m^−2^)	NPP (g·C·m^−2^)
1980s	Summer	0.08	−10.4	−0.01	−3.2	4.20
	Winter	1.68	23.1	−0.06	−54.3	0.84
	Year	0.28	9.9	−0.02	−5.5	−0.10
1990s	Summer	0.42	−122.4	0.05	49.9	6.46
	Winter	−0.70	−18.8	0.09	82.7	−0.02
	Year	0.22	−129.0	0.06	17.81	1.27
2000s	Summer	0.04	107.1	−0.04	−49.3	−9.34
	Winter	−0.82	1.5	0.00	−3.3	0.39
	Year	−0.22	125.5	−0.03	−12.3	−1.11

**Figure 8:**
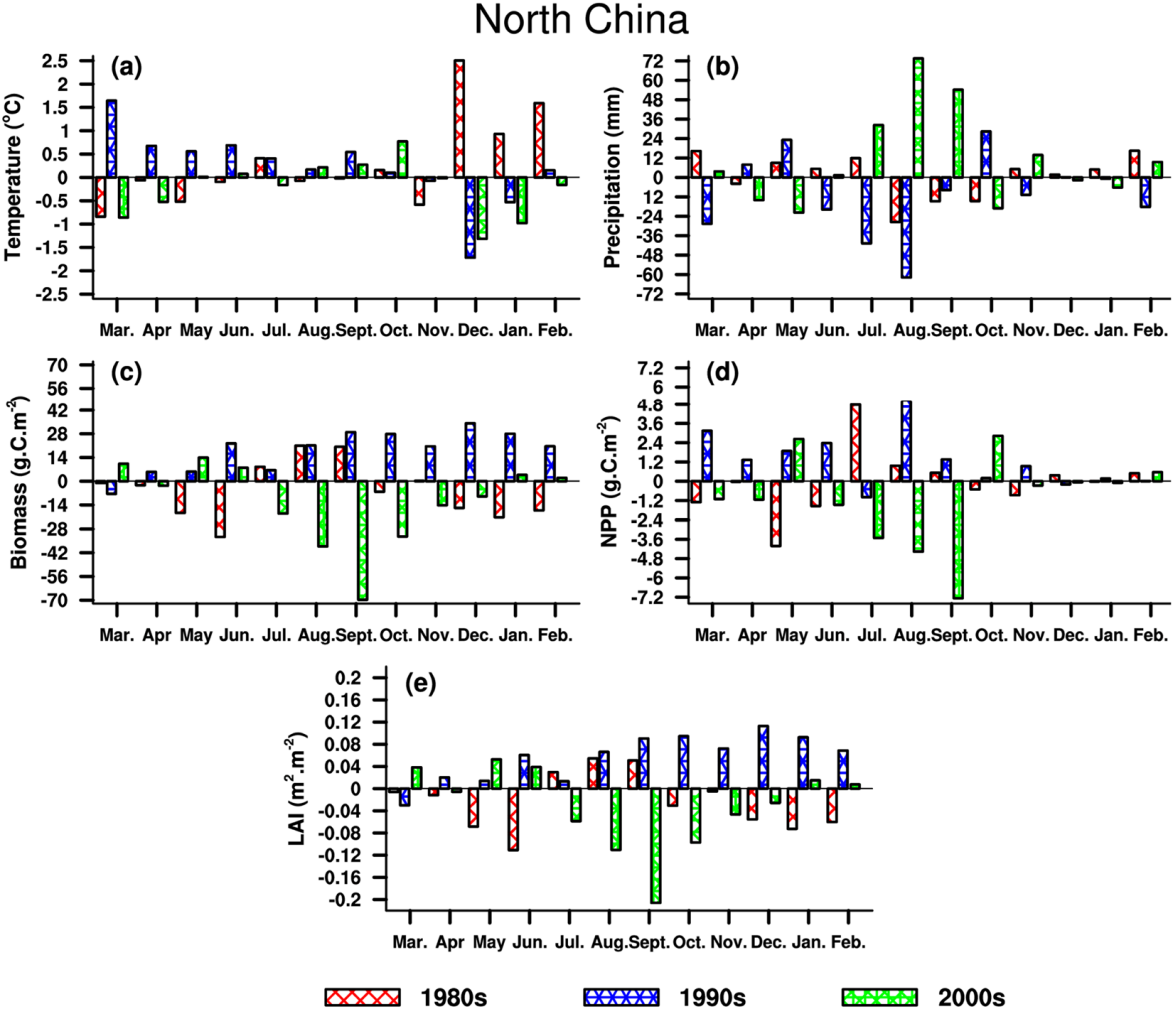
Same as Fig. 7, but for North China. The maps in the figure were created by C.F. using NCL 6.5 (http://www.ncl.ucar.edu/).

It can be seen from Fig. 8 that the changes in wintertime temperature are larger than other seasons, inconsistent with vegetation changes. In the 1980s, a decrease in vegetation is the same as temperature but different from precipitation in most months. However, the larger amplitude of changes in wintertime temperature cannot result in prominent changes in vegetation. In the 1990s, both temperature and vegetation increase, which is opposite to the 1980s. The inconsistent behaviors are found for precipitation in different months, and the opposite changes also occurred in the 1980s. In the 2000s, vegetation demonstrates weaker changes, which might be attributed to the weaker climate change. A decline in LAI and biomass mainly concentrates in summer and autumn while an increase appears in winter and spring, these are not coincident with climate change. NPP exhibits a decline and an opposite behavior occurs for precipitation, except for winter. In general, the vegetation parameters have large discrepancies due to the impacts of hydrothermal conditions in three periods. Nevertheless, NPP shows a different change compared to LAI and biomass in the 2000s, and an analogous change is found around the 1980s.

#### South China

SC belongs to the humid region, where the hydrothermal conditions are beneficial for vegetation growth in four seasons. Table 3 shows that both temperature and precipitation decline (increase) in summer (winter), correspondingly, three vegetation parameters increase in the 1980s. The opposite behaviors are discovered in the 1990s compared to the 1980s, the temperature and precipitation decrease (increase) in winter (summer). Both LAI and biomass increase in summer and winter, but the NPP has a considerable decline in both seasons. In the 2000s, rainfall decreases remarkably, and temperature has a slight increase. Consequently, NPP increases in both seasons, while LAI and biomass decrease (increase) in summer (winter).

**Table 3:** Same as Table 1, but for South China.

Date		Temperature (°C)	Precipitation (mm)	LAI (m^2^·m^−2^)	Biomass (g·C·m^−2^)	NPP (g·C·m^−2^)
1980s	Summer	−0.27	−49.4	0.09	37.8	8.05
	Winter	1.61	16.5	0.07	33.3	12.84
	Year	0.02	−63.2	0.03	4.7	1.12
1990s	Summer	0.10	90.2	0.05	37.3	−4.53
	Winter	−0.63	−17.2	0.05	23.5	−4.02
	Year	0.25	−56.6	0.13	23.12	1.68
2000s	Summer	0.09	−12.3	−0.02	−17.0	0.04
	Winter	0.07	−18.8	0.02	8.3	1.86
	Year	−0.04	−15.8	0.02	1.3	−0.43

**Figure 9:**
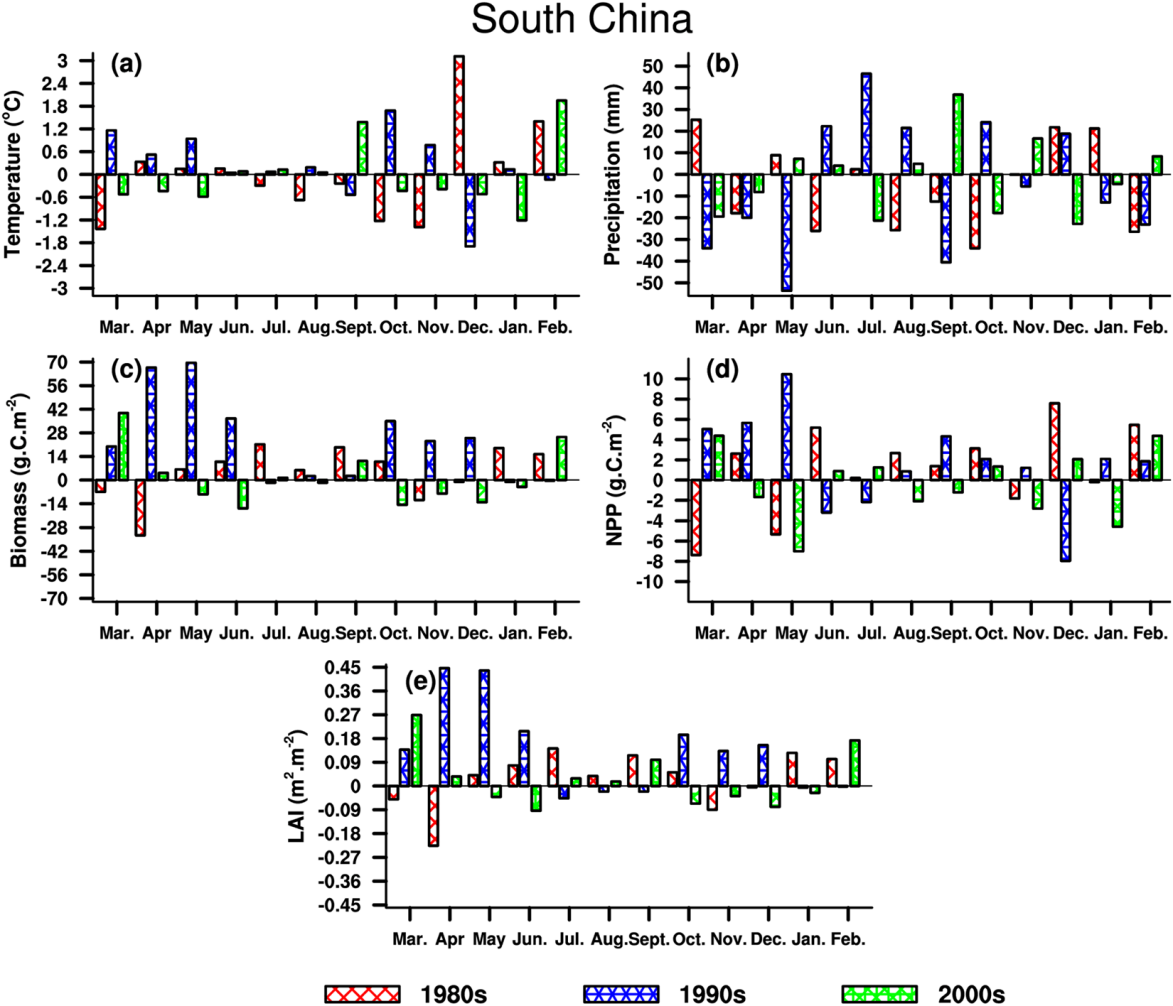
Same as Fig. 7, but for South China. The maps in the figure were created by C.F. using NCL 6.5 (http://www.ncl.ucar.edu/).

Figure 9 illustrates that the changing magnitude of vegetation parameters in spring is larger than other seasons, while these behaviors are not analogous for temperature and precipitation. In the 1980s, the vegetation parameters exhibit the same increase in most months, which is different from the climate change, suggesting that changes in hydrothermal conditions cannot restrict vegetation growth in SC. The vegetation changes around the 1990s are opposite to the 1980s, even though an increase is found in most months. The temperature change is analogous to vegetation behavior in summer half-year, and differs from precipitation. In the 2000s, the changes in LAI and biomass present the consistency with temperature, while the NPP exhibits different responses to climate change. It is clear that the hydrothermal conditions play a minor role to vegetation variations over SC, indicating unfavorable hydrothermal conditions in these regions.

#### Northwest China

NW is located in the arid and semi-arid zones, where vegetation growth is greatly affected by limited rainfall^55^. It can be seen in Table 4 that temperature increases in both summer and winter around the 1980s, while precipitation decreases (increases) in summer (winter). Both LAI and biomass decrease in summer and winter, especially for biomass with a larger amplitude, while NPP exhibits an increase in both seasons. In the 1990s, rainfall increases in both seasons and temperature increases (decreases) in summer (winter). Consequently, LAI and biomass increase both in summer and winter, with an enhanced increase for biomass. NPP has an increase (decrease) in summer (winter). In the 2000s, temperature (precipitation) decreases (increases) in both seasons. There exist no remarkable changes in LAI, while NPP shows a decrease in both seasons, and biomass increases (decreases) in summer (winter).

**Table 4:** Same as Table 1, but for Northwest China.

Date		Temperature (°C)	Precipitation (mm)	LAI (m^2^·m^−2^)	Biomass (g·C·m^−2^)	NPP (g·C·m^−2^)
1980s	Summer	0.21	−3.5	−0.01	−7.1	0.27
	Winter	1.76	8.3	−0.02	−18.9	0.37
	Year	0.34	−7.6	−0.01	−2.1	−0.14
1990s	Summer	0.75	4.7	0.03	22.0	3.12
	Winter	−1.22	6.5	0.04	29.2	−0.13
	Year	−0.05	29.8	0.02	6.52	0.58
2000s	Summer	−0.13	0.3	0.00	1.2	−0.75
	Winter	−1.12	0.6	0.00	−0.1	−0.17
	Year	−0.16	−7.4	0.00	0.3	0.05

**Figure 10:**
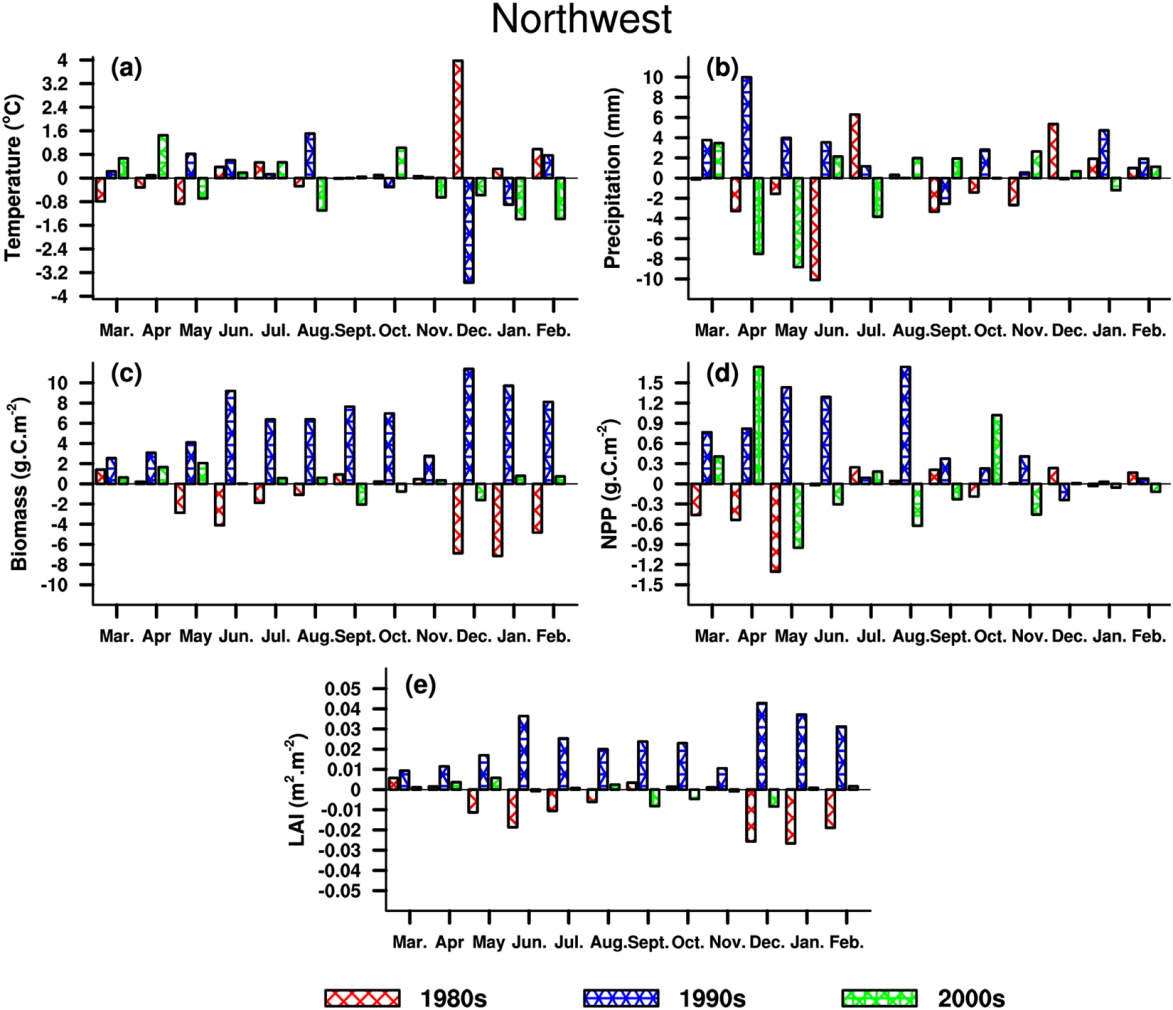
Same as Fig. 7, but for Northwest China. The maps in the figure were created by C.F. using NCL 6.5 (http://www.ncl.ucar.edu/).

Figure 10 illustrates that the amplitude of changes in LAI and biomass are larger in winter, consistent with changes in temperature, while NPP and precipitation exhibit greater changes in spring. The vegetation behaviors are the smallest in NW compared to other three typical subregions, which is related to the dry climatic background. In the 1980s, both LAI and biomass decrease in winter and summer, and the opposite changes occur for temperature. NPP increases significantly with time except for winter, while the same changes appear for precipitation except for autumn. In the 1990s, the vegetation parameters present a synchronous increase in most months, with a similar increase occurring for temperature and precipitation. To some extent, the variations in climate and vegetation indicate the beneficial hydrothermal conditions contributing to vegetation growth in NC. In the 2000s, both LAI and biomass increase significantly with time, except for autumn, this is different from changes in rainfall and temperature. However, NPP change is similar to the temperature in most months. Generally, LAI and biomass always present consistent behaviors over NC, which is analogous to other three typical regions.

#### Comprehensive comparison and analysis

The LAI and biomass always maintain a consistent change during three periods, which is different from the NPP. Nevertheless, the favorable hydrothermal conditions principally contribute to the consistent variability of vegetation in regions where the moisture and heat are the limited factors responsible for vegetation growth. Furthermore, the changes in vegetation parameters also show a similar phenomenon in regions with sufficient water and heat. LAI and biomass are likely to be affected by temperature, while NPP is more susceptible to variations in precipitation, particularly during growing seasons. With the influences from intra-annual variations, the changes in LAI and biomass have larger amplitude from summer to autumn, this is not available for NPP from spring to summer.

Based on the results of regional average shown in Tables 1–4, both climate and vegetation exhibit significantly seasonal and regional characteristics. In the 1980s, wintertime temperature and precipitation increase in four typical subregions, which are different from LAI and biomass but same as NPP. LAI and biomass present a larger variation in winter, excluding SC, while a greater change occurs in summer for NPP. The annual behaviors of vegetation parameters are consistent, except for NC. In the 1990s, the temperature increases (declines) in summer (winter), and the annual rainfall mainly increases in NC and SC. The annual precipitation exhibits an opposite change in comparison with temperature, this coincides with wintertime precipitation.

The summertime precipitation decreases (increases) in NC (SC), this is different from the annual and wintertime precipitation. An increase is found both for LAI and biomass, which is analogous to annual behavior in NPP, while a decline in winter and summer is mainly concentrated in NE and SC. In the 2000s, the temperature decreases (increases) in winter (summer), excluding the SC (NW).

The annual temperature exhibits a warming change except for NE. The precipitation decreases remarkably in NC and SC, but increases in NE. The annual precipitation presents a decline in NW, although a slight increase is found in winter and summer. However, LAI and biomass show opposite changes compared to precipitation in NE and NC, while the NPP behavior is consistent with the precipitation in NE and the temperature in other typical subregions, excluding the annual changes in NW. LAI and biomass share the similar changes in winter, which not occur in other three seasons, and opposite variations in temperature are seen in NE and SC. From the perspective of annual changes, the extreme value of temperature differences is roughly 0.89 °C in the 1980s, and extreme differences of precipitation and vegetation reach up to −129.0 mm, 0.14 m^2^·m^−2^ (LAI), 30.7 g·C·m^−2^ (biomass), and 1.68 m^2^·m^−2^ (NPP), respectively. Climate change has a greater impact on vegetation parameters in the 1990s. Meanwhile, the variations in LAI and biomass are stronger in winter compared to other three seasons, which are different from the NPP changes, indicating that climate change triggers greater influences on LAI and biomass in winter, but on NPP in summer. Moreover, the vegetation parameters always display a consistent behavior in subregions where hydrothermal conditions are adequate or limited.

In addition, the regional and seasonal differences of temperature and precipitation in three periods (Figs. 3 and 4) are also associated with the variability of monsoon system. Around the 1980s, both temperature and precipitation increase in winter, this is closely related to the weakened winter monsoon^56,57^. The amplitude of changes in summertime temperature decreases, while the cooling areas extend due to the weakened summer monsoon^58^, with cyclic increase-decrease pattern in precipitation over East China. In the 1990s, both wintertime temperature and precipitation decrease in East China, associated with enhanced winter monsoon^56^. The increase in temperature is distinct in summer, while the weakened summer monsoon results in increased (decreased) precipitation over South (North) China. In the 2000s, a decrease in wintertime temperature is mainly seen in South China, although the winter monsoon weakened. With the influences of the summer monsoon, the summertime temperature increases over East China, and the precipitation decreases in the mid-lower reaches of YRB and increases over North China. It is needed to be noted that there exists interaction between temperature and precipitation. A warmer atmosphere is capable of holding more moisture^59^, conversely, the reduced precipitation leads to enhanced surface sensible heat and higher temperature^60^. While in-depth analysis of this issue is beyond the scope of our study.

## Conclusions

The regional climate-vegetation coupled model AVIM-RIEMS2.0 is used to study the interaction between climate change and vegetation parameters by incorporating the land use and land cover dataset. The behaviors of LAI, biomass and NPP during three periods are analyzed to examine the impacts of climate change on vegetation carbon flux and morphology over East Asia. The key conclusions are obtained as follows:

1. The effects of climate change on vegetation parameters exhibit evident characteristics at the seasonal and regional scales, due to the differences of hydrothermal conditions caused by climate change. In the 1980s, the temperature and precipitation increase in winter, leading to decrease (increase) in biomass and LAI (NPP). Wintertime precipitation exhibits a cyclic increase-decrease pattern over East China, resulting in an analogous distribution of biomass and LAI. In the 1990s, vegetation parameters show opposite behaviors compared to the 1980s, since temperature and precipitation have opposite changes in comparison with the 1980s. In the 2000s, the changes in wintertime vegetation are restricted by multiple climatic factors in the mid-lower reaches of YRB and southeast coastal regions over China, generating the similar variability of biomass, LAI and precipitation, which cause the consistent variations in NPP and temperature. In summer, vegetation parameters display consistent changes, even though the differences of climate influences appear in various regions.
2. The responses of vegetation parameters to climate change present considerable differences over East Asia. While the biomass and LAI not only show analogous variations, but also have analogous responses to climate change when they are affected by similarly hydrothermal conditions, suggesting the consistent responses of biomass and LAI to regional climate change. However, the responses of NPP to climate change are different from biomass and LAI. Moreover, the responses of vegetation parameters to climate change tend to be homogeneous in regions with plentiful hydrothermal conditions, such as SC.
3. Generally, the vegetation parameters vary with the enhanced amplitude of climate change, and vice versa. For example, the weaker amplitudes of behaviors in biomass, LAI, and NPP are attributed to the weaker variations in temperature and precipitation.
4. There are great differences of the changes in vegetation parameters over three typical subregions. Both biomass and LAI exhibit approximately an opposite change compared to the temperature in NE, NC and NW, while the NPP shows a similar change to temperature, the vegetation parameters exhibit the corresponding changes in SC.

Regional climate change impacts the LULCC through controlling the changes in hydrothermal conditions. Meanwhile, the terrestrial vegetation is affected by climate regimes, since different vegetation types have different requirements for hydrothermal conditions. In this study, the response of land use/cover to climate change is investigated from the point of view of the changes in vegetation carbon flux and morphology. The impacts of climate change on vegetation parameters mainly demonstrate a long-term cumulative effect (e.g. the century scale), it is therefore necessary to conduct model experiments by utilizing long-term land use/cover datasets, while it is not available for us at present. Nevertheless, the effects of climate change on vegetation physiological characteristics are principally reflected at short-term scale. The physiological changes in vegetation parameters have considerable influences on ecological environment. Thus, it is necessary to further assess the effects of regional climate change on vegetation type conversions and vegetation morphology simultaneously in forthcoming studies.
